# After the teleconsultation: getting medicines to patients when pharmacy services are not available

**DOI:** 10.3399/bjgp24X739401

**Published:** 2024-08-30

**Authors:** Rebecca Payne, Archie Lodge, Adam Mackridge, Derek O’Keeffe, Nadia Swann, Aileen Clarke, Jana Schmidt, Thomas Allen, Catherine Sloan, Christine Bond

**Affiliations:** Nuffield Department of Primary Health Care Sciences, University of Oxford, Oxford, UK.; Nuffield Department of Primary Health Care Sciences, University of Oxford, Oxford, UK.; Betsi Cadwaladr University Health Board, Bangor, UK.; School of Medicine, University of Galway, Galway, Ireland.; Nuffield Department of Primary Health Care Sciences, University of Oxford, Oxford, UK.; Nuffield Department of Primary Health Care Sciences, University of Oxford, Oxford, UK.; North Wales GP Out of Hours Service, Betsi Cadwaladr University Health Board, Bangor, UK.; Pharmacist, King Edward Memorial Hospital, Stanley, Falkland Islands.; Royal Flying Doctor Service, Melbourne, Australia.; Institute of Applied Health Sciences, University of Aberdeen, Aberdeen, UK.

## Introduction

It was recognised as early as 1939 that teleconsultations were of limited use if patients were unable to access the medicines recommended on the call.^1^ Nearly a century on, despite a rapid expansion in the remote delivery of consultations, access to urgently required medication for patients remains problematic, with pharmacy and medical services in many areas contracting, particularly during the out-of-hours period and in rural and remote areas. The increased percentage of calls managed purely by telephone,^2^ and the well-documented difficulties finding medical staff willing to work in remote areas,^3^ mean that this challenge has now become a crisis.

Teleconsultations are no longer delivered by radio as they were in 1939, but include telephone and video assessments. They are convenient for patients,^4^ have an excellent safety record,^5^ and enable patients to receive a prompt assessment from home, something particularly valued when people are feeling unwell. They mean clinician time can be used effectively and efficiently, and allow services to be delivered to a wide geographical area by a small cohort of staff, often physically located elsewhere in the region or country. However, this rapid access to clinicians needs to be matched by prompt access to urgently needed medication where necessary. Lack of access can risk morbidity, mortality, poor patient experience, and increased costs further down the patient care pathway. Box 1 provides fictionalised examples of such scenarios.

**Box 1.** Fictionalised examples of challenges accessing medication in the Falkland Islands, Australia, and the UK

Jane Smith is a 45-year-old woman who lives in a small settlement in the south of West Falkland Island, a remote island with fewer than 200 residents and no healthcare facilities. She calls the Falkland Islands’ only emergency department as she has felt unwell for the last 9 days with ear pain, a sore throat, and dizziness. She is advised to obtain clarithromycin tablets from the medical chest located near her and given advice and safety netting over the phone. She is then booked into a face-to-face appointment with the doctor on their next visit to her part of the island. She visits the ‘chest holder’ and is given the medication. The pharmacist then organises re-supply of these medications to the medicine chest holder. They are delivered on a routine flight the next day.
Marie is a 40-year-old farmer who lives 30 minutes’ drive from a small rural Australian town. She has had symptoms of a urinary tract infection for several days. She decides late in the afternoon that she needs to see a GP. She receives a call back from a doctor based in Melbourne. The doctor prescribes antibiotics and suggests that she start taking them immediately. She asks whether she wants an electronic prescription sent to her phone or to the local chemist. As Marie would face a 90-minute drive to collect medication, she delays starting until she can collect more locally the next day.
Seema Patel is an 88-year-old who lives in a small town in the UK. She has become increasingly frail over recent months. Both she and her family believe that she is dying. Her daughter calls 111 on a Friday evening as she is in pain. The out-of-hours GP attends, gives a stat dose of diamorphine, and issues a prescription for further end-of-life medication for the family to collect the next morning. Her son spends the morning driving around nearby pharmacies attempting to source the medication. He returns at 2 pm, by which stage his mother is no longer able to communicate. Seema dies 2 days later. The family feel she was comfortable but her son is deeply distressed that he was not present for the last morning that she was able to communicate.

Realising that there is an urgent need for medication, patients and clinicians often implement expensive, time-consuming, or risky workarounds such as taking a relative’s medication or resorting to a medivac of the patient. Alternatively, many patients delay treatment until the condition worsens. Our team has personally seen the negative effects of this, including unwell patients making long bus, car, or boat journeys to receive urgent medications.

Aside from the obvious medical consequences, there is also a societal factor: poor access to urgently required medication exacerbates existing inequalities, disadvantages rural communities, and discriminates against the poor.^6^ It risks health services breaching national policies such as commitments made in *A Healthier Wales* ^7^ and the *National Strategic Framework for Rural and Remote Health* (Australia).^8^

This article draws on published research, the grey literature, and the lived experience of the author team to outline the different options available for medications access after a teleconsultation when traditional supply routes, for example, through community pharmacy, are not available.

## Current options for medication access after a teleconsultation

Various ways to address this problem have been tried worldwide. These vary in technological sophistication, from basic pharmacy boxes stored in some central place, including anticipatory care medication or rescue packs prescribed to individuals, to medicine chests in remote communities and remote controlled medication vending machines that hold and issue a limited stock of urgently required medications. Some health systems are even moving towards using drones to deliver medication. These potential solutions are shown in Box 2 and described in more detail below.

**Box 2.** Potential solutions for medicines supply

**Solution**	**Advantages**	**Disadvantages**	**Comments**
Anticipatory care packs	Allow tailored end-of-life medication to be put in place in advance by a patient’s own team, who have access to medical notes. Allow rapid administration by community nursing staff when a patient deteriorates	Not always prescribed in advance and can be hard to source during the out-of-hours period. Risk of misuse and diversion	Widely used in the UK
Rescue packs	Allow rapid access to medication for a patient at risk of exacerbation. Avoid unnecessary pressure on the health service for patients who are highly likely to need medication. Encourages personal responsibility for health	Risk of overuse. Risk that patient takes the medication and delays seeking medical help as a result	Widely used in the UK
Medicine chests held by community volunteers	Allow easy access to medicines in remote areas. Facilitate a joined-up teleconsultation service.	Lack of confidentiality. High risk of medicines expiring unused. Require external oversight and audit	Occasional use in remote parts of the UK; regularly used in the Falkland Islands and Australia
Medicines issued by healthcare support workers	Allow robust control of medicine stocks. Can facilitate high-quality teleconsultation (for example, can take observations). Suitable for larger communities	Higher cost. Recruitment challenges to caring roles in the UK. Wage bills may make it unaffordable in high-income settings	Used in Pakistan and tribal areas of North America
Remotely controlled medicines machines	Allow secure and controlled access to medicines in a local community. Facilitate a joined-up teleconsultation service.	Currently unclear if technology will integrate with the workflows within UK out-of-hours services. Limited stock can be held because of capacity. Less suitable for controlled drugs and other items of potential abuse	Used in tribal areas of North America. Currently being trialled in the UK. Medicines and Healthcare products Regulatory Agency allows use by trusts and Health Boards, but current Medicines Act provisions mean additional labelling requirements of the medication are needed if used by community pharmacies
Drones	Can be combined with warehouse automation, which may offer efficient, quick, and low-cost services. Allow access over water, for example, to islands	Travel limits may mean they are unfeasible in some remote, low-population areas	Airspace restrictions may limit use. Unclear UK regulatory position for direct-to-patient deliveries, but already used hospital-to-hospital. Soon to be trialled in the US

## Anticipatory care packs

These packs contain medication needed at the end of life. They are commonly put in place by a patient’s own healthcare provider, such as a GP or hospice service, and held by the patient. Frequently containing painkillers, anxiolytics, anti-emetics, and antisecretory agents, they allow medication to be started in a timely fashion and avoid delays and discomfort.^9^ Medication is issued to an individual patient and is likely to be wasted if they do not require it. Pressures on primary care services or a failure to recognise a deteriorating patient can mean that they are not available for patients who would benefit. As they often contain controlled medications there is a risk of misuse and diversion.^10^ Medication can usually be started by a community professional such as a district nurse following a teleconsultation.

## Rescue packs

Rescue packs are issued in anticipation of an exacerbation of a chronic illness, for example, an infectious exacerbation of chronic obstructive pulmonary disease. They are prescribed to an individual patient and stored in their home. The patient starts them either after a teleconsultation or if they feel their symptoms require it. They allow rapid access to medication for patients at risk of deterioration, but may risk overuse.^11^

## Medicine chests

Medicines (camp) chests are used to provide urgently required medications to small communities in remote areas. In both Australia and the Falkland Islands (Table 1) they are integrated into established healthcare services and provide a standardised set of medications.

**Table 1.** Falkland Islands medicines (camp) chest formulary

Aspirin dispersible tablets 75 mg Buprenorphine s/l tablets 200 µg Codeine phosphate tablets 30 mg Paracetamol suppositories 120 mg Amoxicillin capsules 250 mg Amoxicillin suspension 125 mg/5 ml Clarithromycin suspension125 mg/5 ml Clarithromycin tablets 250 mg Co-amoxiclav 375 mg tablets Flucloxacillin capsules 250 mg Metronidazole tablets 200 mg Chlorphenamine tablets 4 mg Chlorphenamine sugar-free syrup 2 mg/5 ml	Prednisolone tablets 5 mg Salbutamol inhaler 100 µg/puff Spacer device Glyceryl trinitrate spray 400 µg/puff Dioralyte oral powder Omeprazole capsules 20 mg Prochlorperazine buccal tablets 3 mg Sugar and salt spoon Diazepam rectal tube 5 mg Diazepam tablets 2 mg Chloramphenicol 1.0% eye ointment Fluconazole capsule 150 mg Levonorgestrel 1.5 mg

*s/l = sublingual.*

Their supply and operation is underpinned by legislation.^1^^,^^12^ Medicines are held by volunteer members of the community (for example, a farm manager) and are issued from the chest following a telephone consultation with a doctor. Selecting the correct medication is aided by each medicine being assigned a letter of the phonetic alphabet in the Falkland Islands and by a number in Australia.^1^ There are formal systems in place for management of expired medication, audit, and resupply.^13^ They provide a cheap and reliable way of getting medications to patients, but at the risk of confidentiality breaches: although the person issuing the medication is not informed of the clinical indication, they are aware of what medication is issued. Medicine chests traditionally supply very small communities such as a single farm or settlement; the maximum population supplied from one chest in the Falkland Islands is approximately 300 people at Goose Green. This prevents undue burden on the chest holders.

## Medicines issued by healthcare support workers on advice of a doctor, with or without pharmacist involvement

Some healthcare systems rely on healthcare support workers to retrieve medication for the patient from a machine or chest following a teleconsultation.^14^^,^^15^ Although potentially more costly due to the employment of a healthcare support worker, this provides a solution for larger remote communities where volunteers could be overwhelmed by the volume of demand. It may also facilitate a more thorough teleconsultation, with the healthcare support worker able to support the patient with the technology and to take observations such as temperature and heart rate.^14^

## Medicines accessed by the patient from a remotely controlled machine

In remote areas of the US, including North Dakota^16^ and Alaska,^17^ remotely operated medication-issuing machines are situated in communities. Following teleconsultation, the patient is issued with a unique PIN to collect medications from their local machine. Machines typically hold around 20 items of commonly used out-of-hours medication such as antibiotics.^16^ Pilots of similar technology are due to start shortly in North Wales.^18^

## Drones

The use of drones to deliver medication and blood between healthcare sites is well established, with examples in the Isle of Wight^19^ and Rwanda,^20^ where 3400 health centres are supplied by drone flights. Drones also make 75% of rural blood deliveries. Scotland is currently establishing a national drone network, which will include healthcare premises in remote areas.^21^ Recent developments in drone technology combined with warehouse automation allow drones to be used to deliver medications and medical devices directly to the patient’s property, something shortly to be piloted in the US^22^ by the company ‘Zipline’. Older technologies used medications released by parachute from the drone, but modifications now allow a ‘droid’ to be lowered from the drone in order to release the medication on the ground at the exact location required.^22^ There are both regulatory and technical challenges^23^ that need to be addressed in order to use drones to supply medication directly to patients (in contrast with between healthcare sites). There are also potential inequalities issues, for example, with patients with gardens able to receive deliveries, but patients in bedsits harder to serve. This may be less of a barrier in rural areas.

## Discussion

As telemedicine continues to find its place in the delivery of modern health care, telepharmacy has yet to catch up. Low-tech solutions are already widely used, particularly internationally and in rural areas. However, the expansion of telemedicine and the contraction of community pharmacy services in many settings means an expanded population now requires access to medications following a remote consultation. Technological solutions offer promise, but are not ‘plug and play’. Careful evaluation will be needed to ensure that they do not result in unintended consequences such as the exacerbation of existing inequalities, or the erosion of continuity and the introduction of a depersonalised and transactional approach to health care. Much of the current published research on innovative options to supply is restricted to immediate outcome and does not report in depth some of the wider ramifications. There exists a risk that cash-strapped health authorities could use the excuse of a telepharmacy solution to withdraw face-to-face services in remote or hard-to-staff locations. This may result in challenges to the viability of dispensing practices, existing pharmacies, and community hospitals, and make it harder for complex patients less suitable for teleconsultations to access in-person care. Even where technologies work well in one setting, implementing a new technology into a different healthcare system is not as simple as plugging in a new machine. Rather, it requires complex change in organisational workflows, and may not be acceptable to patients and wider stakeholders.

## Conclusion

Although telemedicine has expanded, the systems and processes to get urgently required medication to patients has not kept pace. Exciting new opportunities exist to bring the power of modern technology to solve this problem, but new solutions can cause new and unanticipated problems so further research is needed to ensure safe implementation and an improvement for patients. In order to protect health and save lives it is not enough for health systems to provide teleconsultations; telepharmacy solutions must be brought alongside.
